# Lung Cancer Screening in Individuals With and Without Lung-Related Comorbidities

**DOI:** 10.1001/jamanetworkopen.2022.30146

**Published:** 2022-09-06

**Authors:** Eman M. Metwally, M. Patricia Rivera, Danielle D. Durham, Lindsay Lane, Pasangi Perera, Derek Lamb, Louise M. Henderson

**Affiliations:** 1 Lineberger Cancer Comprehensive Center, University of North Carolina at Chapel Hill; 2 Division of Pulmonary and Critical Care, University of Rochester Medical Center, Rochester, New York; 3 Wilmot Cancer Center, University of Rochester Medical Center, Rochester, New York; 4 Department of Radiology, University of North Carolina at Chapel Hill; 5 Department of Epidemiology, University of North Carolina at Chapel Hill

## Abstract

**Importance:**

Comorbidities characterize the underlying health status of individuals. In the context of lung cancer screening (LCS), lung-related comorbidities may influence the observed benefits and harms.

**Objective:**

To compare the characteristics of individuals undergoing LCS, the LCS examination result, the cancer detection rate (CDR), and the false-positive rate (FPR) in those with and without lung-related comorbidities.

**Design, Setting, and Participants:**

A prospective cohort study was conducted in 5 academic and community screening sites across North Carolina from January 1, 2014, to November 7, 2020. Participants included 611 individuals screened for lung cancer who completed a 1-page health history questionnaire.

**Exposures:**

Presence of at least 1 self-reported lung-related comorbidity, including chronic obstructive pulmonary disease, chronic bronchitis, emphysema, asthma, bronchiectasis, pulmonary fibrosis, silicosis, asbestosis, sarcoidosis, and tuberculosis.

**Main Outcomes and Measures:**

The LCS examination result was determined from the radiologist’s Lung Imaging Reporting and Data System assessment (negative, 1 or 2; positive, 3 or 4). The age-adjusted CDR and FPR were calculated per 100 LCS examinations, using binary logistic regression.

**Results:**

Among the 611 individuals screened for lung cancer (308 men [50.4%]; mean [SD] age, 64 [6.2] years), 335 (54.8%) had at least 1 lung-related comorbidity. Individuals with vs without lung-related comorbidities were more likely to be female than male (180 of 335 [53.7%] vs 123 of 276 [44.6%]; *P* = .02), White vs non-White race (275 of 326 [84.4%] vs 193 of 272 [71.0%]; *P* < .001), and have high school or less education vs greater than a high school education (108 of 231 [46.7%] vs 64 of 208 [30.8%]; *P* = .001). There were no significant differences in the proportion of positive LCS examinations in those with vs without a lung-related comorbidity at baseline (37 [16.0%] vs 22 [11.1%]; *P* = .14) or subsequent (40 [12.3%] vs 23 [10.6%]; *P* = .54) LCS examination. Comparing individuals with vs without lung-related comorbidities, there was no statistically significant difference in the CDR (1.6 vs 1.9 per 100; *P* = .73) or FPR (13.0 vs 9.3 per 100; *P* = .16). Of the 17 individuals with lung cancer, 13 patients (76.5%) were diagnosed with stage I lung cancer.

**Conclusions and Relevance:**

The findings of this study suggest that individuals with self-reported lung-related comorbidities undergoing LCS were more likely to be female, of White race, and have less education than those without lung-related comorbidity. Although no statistically significant differences in the proportion of positive examinations, CDR, or FPR by self-reported lung comorbidities were noted, additional studies with larger numbers of individuals undergoing screening are needed to understand LCS outcomes in those with lung-related comorbidities.

## Key Points

**Question:**

Among individuals undergoing lung cancer screening, do clinical and radiologic findings, cancer detection rate (CDR), and false-positive rate (FPR) differ based on the presence of lung-related comorbidities?

**Findings:**

In this cohort study, more than half of 611 individuals undergoing lung cancer screening had at least 1 lung-related comorbidity; these patients were more likely to be female, of White race, and have a lower educational level. The CDR and FPR were similar among individuals with vs without lung-related comorbidities, and more than 75% of lung cancers diagnosed were stage I non–small cell lung cancer.

**Meaning:**

The findings of this study suggest that the CDR and FPR may be similar regardless of the type of comorbid conditions.

## Teaser

This cohort study compares cancer detection rates and false-positive rates following lung cancer screening in individuals with vs without self-reported lung-related comorbidities.

## Introduction

Lung cancer is the most common cause of cancer-related death worldwide.^1,2^ Lung cancer screening (LCS) with low-dose computed tomography (LDCT) substantially decreases lung cancer mortality^3,4^ and reduces all-cause mortality among high-risk individuals.^4^ In 2021, the US Preventive Services Task Force updated its 2013 recommendations^5^ for annual LDCT screening to include asymptomatic high-risk individuals, defined as those aged 50 to 80 years with at least a 20 pack-year smoking history who currently smoke or quit 15 or more years ago.^6^ Current recommendations do not specify whether LCS should differ among individuals with comorbidities. However, the US Preventive Services Task Force 2021 recommendations state “screening should stop if the individual has a health problem that limits life expectancy or the ability to have lung surgery,” and the shared decision-making conversation should include a discussion of the influence of comorbidities in LCS.^7^

Individuals eligible for LCS have a higher burden of comorbidities than the general population, partly related to their increased smoking intensity and duration. The most commonly reported comorbidities in individuals undergoing LCS are chronic obstructive pulmonary disease (COPD) and cardiovascular diseases.^8,9^ Because comorbidities characterize the underlying health status of individuals, the presence of comorbidities, lung-related comorbidities in particular, may shift the benefits and harms of LCS. Furthermore, lung-related comorbidities, such as emphysema, have been associated with an increased risk of lung cancer.^10^ In addition, depending on the severity of the condition, individuals with lung-related comorbidities may experience increased harm from LCS due to an increased risk of complications associated with procedures and death from causes other than lung cancer.^11,12^

Few studies have assessed lung-related comorbidities and outcomes of LCS in the clinical setting. Therefore, this study sought to compare sociodemographic characteristics, radiologic LCS examination results, cancer detection rate (CDR), and false-positive rate (FPR) among individuals screened for lung cancer with vs without lung-related comorbidities.

## Methods

### Data Sources and Study Population

Data for this study were collected as part of the North Carolina Lung Screening Registry, a National Cancer Institute–funded research registry that prospectively collects data on individuals undergoing LCS as part of routine clinical practice at participating locations throughout North Carolina. Specifically, the North Carolina Lung Screening Registry collects participant sociodemographic and risk factor information from a 1-page risk health history questionnaire (HHQ) and electronic health record (EHR), LCS examination and follow-up information from radiology reports, and outcomes data from the EHR and tumor registry. The HHQ asked individuals to self-report sociodemographic information: educational level, smoking history, comorbidities, family history of lung cancer, and personal history of cancer. From the EHR, we abstracted the following information: age, sex, race and ethnicity, residence, smoking status (current, former, never), body mass index, marital status, and insurance status. These variables were included to characterize the study cohort. From the radiology report in the EHR, we abstracted the Lung Imaging Reporting and Data System (Lung-RADS) assessment and radiologic evidence of emphysema and coronary artery calcifications as noted by the radiologist. We obtained data on lung cancers diagnosed through linkage with an institutional tumor registry. This study was approved by the University of North Carolina Institutional Review Board. All individuals in this study signed an informed consent. We followed the Strengthening the Reporting of Observational Studies in Epidemiology (STROBE) reporting guideline.

We included adults aged 18 years or older undergoing LDCT for LCS between January 1, 2014, and November 7, 2020, at 5 screening sites. This analysis was limited to a subset of individuals who completed the 1-page HHQ at the time of their LCS examination.

### Measurements

#### Exposure

The main exposure was the presence of at least 1 self-reported lung-related comorbidity. Lung-related comorbidities included chronic bronchitis, emphysema, COPD, asthma, bronchiectasis, pulmonary fibrosis, silicosis, asbestosis, sarcoidosis, and tuberculosis.

#### Outcomes

The LCS examination result was classified as positive or negative based on the Lung-RADS assessment.^13^ Negative examinations included those with Lung-RADS of 1 (negative) or 2 (benign appearance). Positive examinations included those with Lung-RADS of 3 (probably benign), 4A (suspicious), or 4B or 4X (highly suspicious). All LCS examinations were followed for 365 days to ascertain whether lung cancer was diagnosed.

The CDR was calculated as the number of positive screening examinations with a pathologic lung cancer diagnosis within 365 days of screening divided by the total number of examinations per 100 examinations. The FPR was calculated as the number of positive screening examinations without a pathologic lung cancer diagnosis within 365 days of screening divided by the total number of examinations per 100 examinations. Among individuals with a lung cancer diagnosis, we categorized histologic findings into non–small cell lung cancer, including adenocarcinoma, squamous cell carcinoma, and adenosquamous cell carcinoma; neuroendocrine carcinoma, including large cell neuroendocrine carcinoma and small-cell lung cancer, and adenoid cystic carcinoma, according to 2015 World Health Organization classification.^14^ Tumor stage was based on the American Joint Committee on Cancer staging system 8th edition.^15^

#### Covariates

Participant residence was categorized as rural or urban using the patient’s zip code and the Rural-Urban Continuum Codes (RUCC), with RUCC 1-3 considered urban and RUCC 4-9 considered rural.^16^ Educational level was based on the highest educational level attained and categorized as high school graduation or less, some education after high school, and college or professional education. Marital status was categorized as married and unmarried (never married, widowed, separated, or divorced). Smoking status was classified as current, former, or never. Insurance types included Medicare and Medicare Advantage, Medicaid, private, and uninsured. Body mass index (calculated as weight in kilograms divided by height in meters squared) was categorized as underweight or normal weight (<25) and overweight or obese (≥25). First-degree family history of cancer was categorized as yes if any of the following were reported: mother, father, brother, sister, or child. Prior lung biopsy was based on EHR abstraction as yes or no. The number of LCS examinations was based on data extracted from the EHR.

### Statistical Analysis

We compared the demographic, self-reported comorbid conditions, and clinical characteristics among those with vs without self-reported lung-related comorbidities using χ^2^ tests and the Fisher exact test. We reported the counts and proportion of missing data. We also compared the proportion of negative vs positive LCS results at baseline and subsequent screening in individuals with vs without lung-related comorbidities using a χ^2^ test. The CDR and FPR were calculated per 100 screening examinations using age-adjusted binary logistic regression. Analyses were conducted using SAS, version 9.4 (SAS Institute Inc). Using unpaired, 2-sided testing, the significance threshold was α = .05.

## Results

This study of 611 individuals who underwent LCS included 303 women (49.6%) and 308 men (50.4%); mean (SD) age was 64 (6.2) years. A total of 335 participants (54.8%) reported at least 1 lung-related comorbidity and 276 (45.2%) reported no lung-related comorbidities. Individuals with vs those without lung-related comorbidities were more likely to be female than male (180 of 335 [53.7%] vs 123 of 276 [44.6%]; *P* = .02), White vs non-White race (275 of 326 [84.4%] vs 193 of 272 [71.0%]; *P* < .001), or have a high school or less educational level (108 of 231 [46.7%] vs 64 of 208 [30.8%]; *P* = .001) (Table 1). There were no significant differences in self-reporting of non–lung-related comorbidities between individuals with vs without lung-related comorbidities. Of 611 individuals, the most commonly reported non–lung-related comorbidities were hypertension (227 [37.2%]), diabetes (128 [21.0%]), heart disease (112 [18.3%]), history of cancer (109 [17.8%]), and stroke (49 [8.0%]). Radiologic findings of emphysema on LDCT were reported more often in individuals with vs without self-reported lung-related comorbidities (178 [53.1%] vs 91 [33.0%]; *P* < .001). Coronary artery calcification was noted in the radiologist report in 382 (41.2%) individuals screened. On the LDCT report, coronary artery calcification was similar between individuals with vs without lung-related comorbidities (207 [61.8%] vs 175 [63.4%]). There were no statistically significant differences in self-report of heart disease between the 2 groups. Of 335 individuals who self-reported lung-related comorbidities, COPD, emphysema, and/or chronic bronchitis were reported by 83.3%, emphysema or chronic bronchitis by 22.4%, bronchial asthma by 26.6%, bronchiectasis by 9%, lung fibrosis by 1.8%, sarcoidosis by 1.8%, tuberculosis by 1.5%, and asbestosis by 0.3% (Figure).

**Table 1.** Characteristics of Individuals Undergoing Lung Cancer Screening by Self-reported Lung-Related Comorbidity

Characteristic^a^	No. (%)			*P* value
	All (N = 611)	Self-reported lung-related comorbidity (n = 335)	No self-reported lung-related comorbidity (n = 276)	
Age, y				
<54	13 (2.1)	8 (2.4)	5 (1.8)	<.01
55-59	146 (23.9)	94 (28.1)	52 (18.8)	
60-64	181 (29.6)	83 (24.8)	98 (35.5)	
65-69	120 (19.6)	57 (17.0)	63 (22.8)	
70-74	114 (18.7)	67 (20.0)	47 (17.0)	
75-80	37 (6.1)	26 (7.8)	11 (4.0)	
Sex				
Female	303 (49.6)	180 (53.7)	123 (44.6)	.02
Male	308 (50.4)	155 (46.3)	153 (55.4)	
Race				
Black	119 (19.9)	47 (14.4)	72 (26.5)	<.001
White	468 (78.3)	275 (84.4)	193 (71.0)	
Other^b^	11 (1.8)	4 (1.2)	7 (2.6)	
Missing^c^	13 (2.1)	9 (2.7)	4 (1.5)	
Smoking^d^				
Current	284 (46.8)	147 (44.1)	137 (50.0)	.16
Former	322 (53.0)	185 (55.6)	137 (50.0)	
Never	1 (0.2)	1 (0.3)	0	
Missing^c^	4 (0.7)	2 (0.6)	2 (0.7)	
Educational level				
<HS or HS graduate	172 (39.2)	108 (46.8)	64 (30.8)	.001
Some post-HS	149 (33.9)	78 (33.8)	71 (34.1)	
College/professional	118 (26.9)	45 (19.5)	73 (35.1)	
Missing^c^	172 (28.2)	104 (31.0)	68 (24.6)	
Residence				
Urban	496 (81.2)	269 (80.3)	227 (82.2)	.54
Rural	115 (18.8)	66 (19.7)	49 (17.8)	
Insurance				
Medicare/Medicare Advantage	375 (62.7)	214 (64.9)	161 (60.1)	.12
Any Medicaid	52 (8.7)	32 (9.7)	20 (7.5)	
Any private	128 (21.4)	59 (17.9)	69 (25.7)	
Uninsured	43 (7.2)	25 (7.6)	18 (6.7)	
Missing^c^	13 (2.1)	5 (1.5)	8 (2.9)	
Marital status				
Married	292 (49.6)	155 (48.0)	137 (51.5)	.47
Unmarried	297 (50.4)	168 (52.0)	129 (48.5)	
Missing^c^	22 (3.6)	12 (3.6)	10 (3.6)	
BMI				
Underweight/normal	184 (30.5)	108 (32.4)	76 (28.1)	.23
Overweight/obese	419 (69.5)	225 (67.6)	194 (71.9)	
Missing^c^	8 (1.3)	2 (0.6)	6 (2.2)	
Non–lung-related comorbidities^e^				
Hypertension	227 (37.2)	127 (37.9)	100 (36.2)	.67
Diabetes	128 (21.0)	71 (21.2)	57 (20.7)	.87
Heart disease	112 (18.3)	69 (20.6)	43 (15.6)	.11
Prior cancer diagnosis	109 (17.8)	54 (16.1)	55 (19.9)	.22
HIV	19 (3.1)	7 (2.1)	12 (4.3)	.11
Stroke	49 (8.0)	24 (7.2)	25 (9.1)	.39
Family history of lung cancer				
Yes	157 (37.9)	95 (41.3)	62 (33.7)	.08
No	257 (62.1)	135 (58.7)	122 (66.3)	
Missing^c^	197 (32.2)	105 (31.3)	92 (33.3)	
History of cancer				
Yes	109 (17.8)	54 (16.1)	55 (19.9)	.22
No	502 (82.2)	281 (83.9)	221 (80.1)	
Prior lung biopsy or surgery				
Yes	32 (5.2)	22 (6.6)	10 (3.6)	.10
No	579 (94.8)	313 (93.4)	266 (96.4)	
No. of lung cancer screening examinations				
1	342 (56.0)	175 (52.2)	167 (60.5)	.15
≥2	269 (44.0)	160 (41.8)	109 (39.5)	
Emphysema on LDCT	269 (44.0)	178 (53.1)	91 (33.0)	<.001
CAC on LDCT	382 (41.2)	207 (43.0)	175 (63.4)	.19

Abbreviations: BMI, body mass index (calculated as weight in kilograms divided by height in meters squared); CAC, coronary artery calcification; HS, high school; LDCT, low-dose computed tomography.

^a^ Characteristics of individuals collected during the screening visit.

^b^ Includes Asian individuals and those who selected more than one race.

^c^ Missing is not included in the column percentages or χ^2^ calculations.

^d^ Current vs former smokers.

^e^ Individuals could report more than one comorbid condition.

**Figure.**
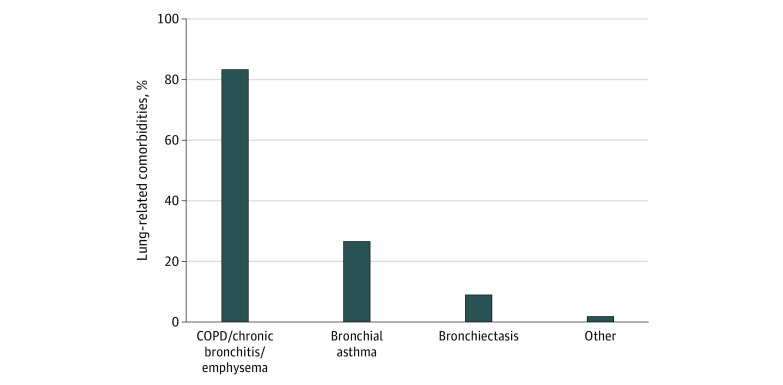
Distribution of Lung-Related Comorbidities Among 335 Individuals COPD indicates chronic obstructive pulmonary disease.

Of the 971 LCS examinations, 429 (44.2%) were baseline screenings and 542 (55.8%) were subsequent screenings (Table 2). Positive LCS examinations constituted 59 (13.8%) of baseline vs 63 (11.6%) of subsequent examinations. Individuals with vs without lung-related comorbidities had similar proportions of positive examinations at baseline (37 [16.0%] vs 22 [11.1%]; *P* = .14) and on subsequent screening examinations (40 [12.3%] vs 23 [10.6%]; *P* = .54). The FPR was similar between individuals with vs without lung-related comorbidities (13.0 vs 9.3 per 100; *P* = .16). The overall CDR was 1.8 per 100 screening examinations and was similar (1.6 vs 1.9 per 100; *P* = .73) between individuals with vs without lung-related comorbidities.

**Table 2.** Radiologic Findings and Screening Outcomes of Screening Examinations of Those With vs Without Self-reported Lung-Related Comorbidities

Characteristics	No. (%)			*P* value
	All examinations (N = 971)^a^	Self-reported lung-related comorbidity (n = 556)	No self-reported lung-related comorbidity (n = 415)	
Lung-RADS				
Baseline				
No.	429	231	198	
Negative	370 (86.2)	194 (84.0)	176 (88.9)	.14
Positive	59 (13.8)	37 (16.0)	22 (11.1)	
Subsequent				
No.	542	325	217	
Negative	479 (88.4)	285 (87.7)	194 (89.4)	.54
Positive	63 (11.6)	40 (12.3)	23 (10.6)	
Lung cancer				
No. of individuals	17	9	8	
CDR per 100^b^	1.8	1.6	1.9	.73
False-positive				
No. of individuals	105	68	37	
FPR per 100^b^	11.2	13.0	9.3	.16

Abbreviations: CDR, cancer detection rate; FPR, false-positive rate; Lung-RADS, Lung Imaging Reporting and Data System.

^a^ Number of screening examinations; some individuals had more than 1 examination.

^b^ Calculated per 100 screening examinations using age-adjusted binary logistic regression.

Of 611 individuals enrolled in this study, 17 were diagnosed with lung cancer through screening: most were younger than 65 years (11 [64.7%]). Most lung cancer was diagnosed on baseline screening (13 [76.5%]), was non–small cell lung cancer (14 [82.4%]), and was categorized as stage I (13 [81.3%]) (Table 3). Adenocarcinoma was the most common histologic type (11 of 17 [64.7%]). All adenocarcinoma diagnosed was pathologically defined as invasive carcinoma per the World Health Organization classification of lung tumors except for 2 cases of minimally invasive adenocarcinoma and adenocarcinoma in situ.^14^ In addition, there were 2 cases of neuroendocrine tumors: large-cell neuroendocrine and small-cell lung cancer.

**Table 3.** Characteristics of Individuals With Screen-Detected Lung Cancer

Characteristic	No. (%)		
	Total (N = 17)	Self-reported lung-related comorbidity (n = 9)	No self-reported lung-related comorbidity (n = 8)
Age, y			
<65	11 (64.7)	6 (66.7)	5 (62.5)
≥65	6 (35.3)	3 (33.3)	3 (37.5)
Sex			
Male	9 (52.9)	7 (77.8)	2 (25.0)
Female	8 (47.1)	2 (22.2)	6 (75.0)
Race			
White	12 (70.6)	7 (77.8)	5 (62.5)
Black	5 (29.4)	2 (22.2)	3 (37.5)
Examination type			
Baseline	13 (76.5)	7 (77.8)	6 (75.0)
Subsequent	4 (23.5)	2 (22.2)	2 (25.0)
Lung-RADS assessment^a^			
3	1 (5.9)	1 (11.1)	0
4A	7 (41.2)	5 (55.6)	2 (25.0)
4B	5 (29.4)	2 (22.2)	3 (37.5)
4X	4 (23.5)	1 (11.1)	3 (37.5)
Histopathologic findings			
NSCLC	14 (82.4)	8 (88.9)	6 (75.0)
Neuroendocrine tumor	2 (11.8)	1 (11.1)	1 (12.5)
Adenoid cystic carcinoma	1 (5.9)	0	1 (12.5)
Staging^b^			
I	13 (81.3)	8 (88.9)	5 (71.4)
II	0	0	0
III	1 (6.3)	0	1 (14.3)
IV	1 (6.3)	1 (11.1)	0
Limited-stage SCLC	1 (6.3)	0	1 (14.3)
Missing	1 (5.9)	0	1 (12.5)

Abbreviations: Lung-RADS, Lung Imaging Reporting and Data System; NSCLC, non–small cell lung cancer; SCLC, small-cell lung cancer.

^a^ Assessment scale: 3, probably benign; 4A, suspicious; and 4B or 4X, highly suspicious.

^b^ Percentages based on the number of responses vs the full cohort; missing values are reported as the number and percentage missing and are not included in the column percentages or χ^2^ calculations.

## Discussion

In this study, approximately half of individuals undergoing LCS reported at least 1 lung-related comorbidity. Individuals with self-reported lung-related comorbidities were more likely to be female vs male, White vs non-White, and have less educational attainment than individuals without lung-related comorbidities. There were no significant differences in positive LCS examinations, CDR, or FPR based on the presence of lung-related comorbidity. Most screen-detected lung cancers were identified in individuals younger than 65 years and were stage I non–small cell lung cancer.

The study cohort had higher frequencies of almost all self-reported comorbidities vs the National Lung Screening Trial (NLST) cohort^17^: COPD (45.7% vs 17.5%), asthma (14.6%, vs 6.2%), heart disease (18.3% vs 12.7%), diabetes (21.0% vs 9.7%), history of cancer (17.8% vs 4.2%), and stroke (8.0% vs 2.8%). The study cohort was similar to NLST in terms of hypertension (37.2% vs 35.4%), current smoking status (46.8% vs 48.2%), and overweight/obesity (69.5% vs 70.5%). In addition, the study cohort was older compared with the NLST (44.4% vs 26.6% aged ≥65 years), with a higher proportion of female (49.6% vs 41.0%), Black (19.9% vs 4.5%), and unmarried (50.4% vs 33.3%) individuals. Similar to our findings, earlier studies have shown that individuals undergoing LCS in clinical settings have a higher comorbidity burden than participants enrolled in the NLST.^18,19,20^ At an urban safety-net hospital in Boston, 1203 individuals undergoing LCS had higher frequencies of comorbidities than those in NLST, including COPD (36.0% vs 17.5%), asthma (18.6% vs 6.2%), heart disease (17.6% vs 13.0%), hypertension (62.6% vs 35.2%), diabetes (25.9% vs 9.7%), and history of cancer (9.2% vs 4.0%).^18^ In another study of a single New York academic LCS program, 1181 individuals had higher frequency of comorbidities: chronic lung disease (67%), hypertension (67%), diabetes (34%), and heart disease (17%).^19^ A retrospective analysis of the Kaiser Permanente Colorado database reported that 3375 individuals undergoing LCS had more lung-related comorbidities compared with NLST (32.5% vs 17.5%; *P* < .001).^20^ The higher prevalence of comorbidities during LCS at the population level compared with NLST warrant incorporation of comorbidities into screening decisions because they affect general health status and, based on severity, may affect the balance of risks and benefits observed in the NLST.^21^

We did not find statistically significant differences in screening outcomes between individuals with vs without lung-related comorbidities regarding positive examinations at baseline (16.0% vs 11.1%; *P* = .14) or subsequent times (12.3% vs 10.6%; *P* = .54), CDR (1.6 vs 1.9 per 100; *P* = .73) or FPR (13.0 vs 9.3 per 100; *P* = .16). In a retrospective analysis of 3375 individuals undergoing LCS at Kaiser Permanente Colorado, COPD was significantly associated with increased odds of positive examinations adjusted for other covariates (odds ratio, 1.35; 95% CI, 1.07-1.71).^20^ An analysis of the NLST participants found COPD was associated with an increased CDR and notably higher procedure rate and procedure-related complications.^22^ In a study of 5835 LCS examinations across a population network in Boston, CDR was 2%, FPR was 13%, and COPD was 1 of the significant predictors of increased FPR, in addition to patient age, baseline scan, and radiologist experience.^23^ In our study, we examined a relatively small sample size and evaluated COPD as one of several self-reported lung-related comorbidities, compared with other studies^20,22,23^ that had a larger sample size and evaluated COPD as the only comorbidity. In addition, COPD diagnosis is challenging^24,25^ because it is frequently underreported in the settings of LCS.^26,27^ Our study population included 7.2% participants without health insurance. This group may be more likely to postpone follow-up care after a positive LCS finding, leading to a lower observed rate of cancer detection and a higher rate of false-positive reports within 365 days. These factors might partially explain why we did not find differences in LCS outcomes based on the type of comorbidity compared with others.^20,22,23^

In our study, 17 of 611 individuals had screen-detected lung cancer. Most patients were younger than 65 years, diagnosed at baseline LDCT with stage I non–small cell lung cancer, more commonly adenocarcinoma. Lung-related comorbidities were prevalent among 9 of 17 patients, but because of the small number of participants, we did not examine differences regarding sociodemographic, radiologic, or histologic characteristics, or stage at diagnosis among patients with screen-detected lung cancer based on the type of comorbidity. In a post hoc analysis of NLST, participants in the LDCT arm with COPD based on spirometric data, had lower rates of overdiagnosis (defined as carcinoma in situ and minimally invasive bronchioalveolar carcinoma–related cancer) and a more favorable stage shift (defined as increased early-stage and decreased late-stage diagnosis) compared with patients with no airflow limitation, after correction for overdiagnosis.^28^ These findings echo the importance of addressing lung-related comorbidities and their severity during LCS to balance the benefits of early-stage diagnosis with potential complications related to underlying comorbidities.

### Strengths and Limitations

This study has strengths and limitations. We included data from multiple academic and community practices representing clinical LCS settings. We collected detailed information about lung cancer risk factors by abstracting data from EHRs and through the HHQ. Limitations of our study include the possibility of loss to follow-up if individuals sought medical care elsewhere. However, we linked individuals' records to outcome data from an institutional tumor registry. A second limitation was the lack of data on comorbidity severity, which may provide additional information on the association of comorbidities with LCS outcomes. A third limitation is the use of self-reported lung-related comorbidities as our main exposure without incorporating pulmonary function tests. However, other large studies have used self-reported comorbidities, including the NLST.^17,29^ In addition, the most common lung-related comorbidity (COPD) may have been underdiagnosed or overdiagnosed in the study cohort, as reported elsewhere.^26,27,30^

## Conclusions

In this study, comorbidities, especially lung-related comorbidities, were common among individuals undergoing LCS. Those with vs without lung-related comorbidities were more likely to be female, White, and have less education. Unlike earlier studies, we found similar rates of positive LDCT examinations, CDR, and FPR between individuals with and without lung-related comorbidities. Future studies are needed to pool evidence from clinical settings about the association between comorbidities and LCS outcomes and implement standardized measures for incorporating comorbidities in screening decisions.
